# *Senna petersiana* (Bolle) Lock: A Review of Its Ethnomedicinal Uses, Phytochemistry, Pharmacological Activities, and Toxicological Profile

**DOI:** 10.3390/plants14243800

**Published:** 2025-12-13

**Authors:** Talita Jessica Mnisi, Mashilo Mash Matotoka, Peter Masoko

**Affiliations:** Department of Biochemistry, Microbiology, and Biotechnology, University of Limpopo, Private Bag X1106, Sovenga 0727, South Africa; talita.mnisi@ul.ac.za (T.J.M.); mashilo.matotoka@ul.ac.za (M.M.M.)

**Keywords:** *Senna petersiana*, ethnomedicinal, phytochemistry, anti-virulence, antibacterial, toxicology

## Abstract

*Senna petersiana* (Bolle) Lock is a chemically diverse plant widely recognized for its ethnomedicinal applications across various traditional medical systems. It is native to and widely distributed in African countries, including Ethiopia, Cameroon, and South Africa. This review integrates the phytochemical composition, biological activities, and toxicological effects of *S. petersiana*. Phytochemical analyses reveal the presence of numerous classes of compounds, including alkaloids, flavonoids, phenolics, anthraquinones, chromones, and sterol glycosides, with variations in concentration across different plant parts. Quantitative studies highlight particularly high levels of phenolics and flavonoids in ethanol, methanol, and acetone extracts, correlating these with enhanced biological activities. Pharmacological investigations demonstrate a spectrum of activities, including antibacterial, antioxidant, anti-inflammatory, antiviral, anthelmintic, and anticancer effects, supporting many of the plant’s traditional uses. Toxicological assessments suggest relative safety at moderate doses, though further evaluation is necessary for specific cell types and high-dose exposures. Despite the promising bioactivities, the mechanisms of action and in vivo efficacy of isolated compounds remain underexplored. Future research should focus on bioassay-guided isolation, detailed pharmacodynamic studies, and comprehensive toxicological profiling to validate and harness the therapeutic potential of *S. petersiana*. This review highlights the plant’s biochemical complexity and paves the way for its development as a valuable phytopharmaceutical agent.

## 1. Introduction

Traditionally, medicinal plants have remained an integral part of healing in many developing countries, where some places rely exclusively on this system for their primary health care needs [1]. Due to their cultural value and cost-effectiveness worldwide, these plants are used as an alternative treatment for a variety of medical conditions [2]. Medicinal plants have enormous potential for scientific research, as they harbour numerous phytochemicals that may yield novel pharmaceutical compounds [3]. Due to being rich in medicinal compounds, the fact that antimicrobial resistance is on the rise, and the increase in bacterial infections, medicinal plants are considered a vital resource for new drug development and alternative therapeutics [4].

*Senna petersiana* (Bolle) Lock is a species of the genus *Senna,* a large and taxonomically complex group within the family Fabaceae [5]. The genus comprises approximately 600 species, predominantly distributed in tropical and subtropical regions of the world [6]. Members of the *Senna* genus have long been recognized for their medicinal, ornamental and ecological significances. A number of species, such as *Senna alexandrina* Mill. and *Senna occidentalis* (L.) Link have been well investigated, and their laxative capability, microbial and antioxidant properties have been reported [7].

*S. petersiana* (syn. *Cassia petersiana* Bolle) is a flowering plant in the kingdom Plantae, class Magnoliopsida (dicotyledons), order Fabales, family Fabaceae, and subfamily Caesalpinioideae. It is a deciduous shrub or small tree native to various African countries. It is widely distributed across countries, including Ethiopia, Cameroon, Madagascar and South Africa [8]. The plant is commonly known as eared *senna*, monkey pod, Munembenembe, and Uhwabile [9]. It features compound leaves with 6–12 pairs of opposite leaflets and produces bright yellow, sweetly scented flowers arranged in large sprays. When mature, it yields elongated, slightly curved pods up to 25 cm long, which are consumed locally, either fresh or prepared as a porridge-like dish [10]. *S. petersiana* is indigenous to sub-Saharan Africa and grows in a wide range of habitats, from woodlands and savannas to riverbanks and grasslands [11]. *S. petersiana* is classified as least concern on the Red List of South African Plants, indicating that it is currently widespread and abundant across its natural range and does not face any significant immediate threat of extinction [12].

Despite belonging to a widely studied genus, *S. petersiana* is still a relatively understudied plant on its phytochemistry, pharmacological activities, and traditional uses. Several *Senna* species such as *S. occidentalis* [13], and *S. alexandrina* [14] have been extensively investigated and reviewed for their phytochemistry and biological activities highlighting the knowledge gap for *S. petersiana*. Therefore, this review aims to gather existing, yet fragmented, research on its botanical characteristics, traditional medicinal uses, phytochemical composition, and pharmacological effects. Given the evidence presented, this study aims to emphasize the plant’s potential applications, provide directions for future research questions, and elucidate the connections between its ethnomedicinal use and modern pharmacological findings.

## 2. Methodology

To compile a comprehensive evidence base, we systematically searched major bibliographic databases, including PubMed, ScienceDirect, Web of Science, and Google Scholar, and complemented these searches with dissertations and theses. The search strategy combined the accepted scientific name and known synonyms, including “*Senna petersiana*,” “*Cassia petersiana*,” “*S. petersiana*,” and related phrases such as “*Senna petersiana* extract,” “*Senna petersiana* phytochemistry,” “*Senna petersiana* antioxidant,” “*Senna petersiana* antimicrobial,” “*Senna petersiana* toxicity,” and “ethnomedicinal and *Senna petersiana*”. We included studies published up to August 2025, spanning in vitro and in vivo investigations, ethnobotanical surveys, chemical profiling, antioxidant assays, antimicrobial activity, and safety evaluations. Review articles, studies outside the scope of the review, articles lacking primary data, and conference abstracts without full results were excluded.

## 3. Ethnomedicinal Uses

Plants in the *Senna* genus are well-known for their traditional medicinal uses across many parts of the world. For example, *Senna alexandrina* Mill. is well known worldwide as a natural laxative [5], while species such as *S. occidentalis* and *Senna siamea* (Lam.) H.S.Irwin and Barneby have long been used in Asia to treat infections, fever, and digestive problems [15,16,17,18]. These species show that, while each has its unique uses, there is often some overlap in how different parts of the plants are applied.

Within this diverse genus, *S. petersiana* stands out in sub-Saharan African ethnobotany for its multi-part medicinal applications. Indigenous communities use various parts of the plant, including roots, leaves, bark, and sometimes seeds, to treat a wide range of ailments. These practices vary among ethnic groups and regions, often influenced by ecological availability and cultural beliefs [19,20]. The ethnomedicinal uses of the plant are mentioned below and in Table 1.

### 3.1. The Use of the Whole Plant

When the plant is used as a whole (including stems, leaves, roots, and bark), it is commonly prepared as a decoction or cold maceration and then administered both orally and topically, particularly through therapeutic bathing [21,22]. In traditional applications, such whole plant decoctions are used for convulsions, bone pain, arthritis, diabetes, cholera, and fever [22]. This approach reflects the broader ethnomedicinal practice of using all plant parts synergistically to enhance therapeutic efficacy [23].

However, compared with other species in the *Senna* genus, there is considerable diversity in preparation methods and therapeutic applications, primarily influenced by geographical, cultural, and phytochemical factors. For instance, in India, the whole plant of *Senna angustifolia* (Vahl) H.S.Irwin and Barneby is primarily used for its well-known laxative properties, mainly due to the presence of anthraquinone glycosides such as sennosides [24]. This contrasts with the use of *S. petersiana* as a whole, where laxative effects are not prominently reported, suggesting a different phytochemical profile or a different traditional medical framework in African systems. Similarly, in various parts of Asia, species such as *Senna obtusifolia* (L.) H.S.Irwin and Barneby and *S. occidentalis* are also utilized in whole plant form. Traditional uses in these regions include treatments for ophthalmic disorders, urinary tract issues, constipation, and infectious diseases such as gonorrhea and tuberculosis [25,26,27].

These contrasts in therapeutic applications suggest that while whole plant use is a shared practice across the genus, the specific cultural, ecological, and possibly phytochemical contexts determine how each species is applied. In Africa, *S. petersiana* is used primarily for infectious conditions, whereas in Asia, the focus for other *Senna* species tends toward gastrointestinal, ophthalmic, and urinary conditions [22,25,26,27]. This highlights the versatility of the genus in ethnomedicine, as well as the adaptability of whole plant usage based on local health priorities and empirical knowledge systems.

### 3.2. Ethnobotanical Uses of S. petersiana Roots

Among the most frequently used parts of *S. petersiana*, the roots hold a prominent place in traditional medicine. They are boiled to make decoctions or warm infusions, taken orally to treat ailments such as stomach complaints, constipation, sexually transmitted infections, malaria, intestinal parasites, bilharzia, and skin infections [19,20,28,29]. Root use extends into ethnoveterinary practice, where ground roots mixed in water are used to treat red water disease in cattle [30], underlining their cultural and practical importance in both human and animal health.

Roots are also widely used across other *Senna* species. For example, *S. rugosa* in Brazil is used as an anthelmintic remedy and a snakebite antidote [31], while *Senna podocarpa* (Guill. and Perr.) Lock, native to Africa, is used to treat gonorrhea and skin ailments [32]. These uses are similar to those of *S. petersiana*, particularly in the management of infections and inflammatory conditions. Some species, however, show more distinct applications. In India, *Senna auriculata* (L.) Roxb. roots are used for rheumatism and eye diseases [33], suggesting a shift toward musculoskeletal and sensory conditions. Notably, *Senna velutina* (Vogel) H.S.Irwin and Barneby in Brazil is reportedly used to treat leukemia [34]. These examples reinforce the idea that roots are central to traditional *Senna* use worldwide, though local knowledge systems guide the specifics. *S. petersiana* roots, while not uniquely specialized, represent a generalist therapeutic tool, widely applied across multiple disease categories.

### 3.3. The Different Ethnobotanical Uses of S. petersiana Leaves

Leaf use in *S. petersiana* is versatile. Decoctions made from the leaves are prepared by boiling or soaking, then consumed for a wide range of conditions, including malaria, typhoid, hepatitis, intestinal worms, skin infections, vomiting, loss of appetite, and even syphilis [6,10,19,25,30,35]. In some cultural contexts, leaves are also included in steam baths or applied externally, particularly for treating skin diseases and breast pain [6], indicating both internal and external therapeutic use.

What makes the leaf particularly interesting across the genus is the range of functions it assumes. In West Africa, the leaves of *Senna spectabilis* (DC.) H.S.Irwin and Barneby are used for skin lesions and labour pain relief, suggesting a specialized role in maternal care [36,37]. Meanwhile, in China and Cameroon, *Senna* leaves are turned to for issues like insomnia, epilepsy, anxiety, and skin conditions such as eczema and scabies [38,39]. These neurological and dermatological indications differ from the infection- and digestion-focused uses in African traditions.

In Mexico, *Senna septemtrionalis* (Viv.) H.S.Irwin and Barneby leaves are used in conditions ranging from alopecia to rabies and snakebite [40,41]. This wide variety across regions suggests that the leaf is not only accessible but also pharmacologically dynamic. Within this context, the broad spectrum of infectious diseases, including gastrointestinal and inflammatory conditions, observed in *S. petersiana* leaves aligns with the genus trend while maintaining regional specificity.

### 3.4. Recorded Uses of S. petersiana Seeds

Unlike the roots or leaves, the seeds of *S. petersiana* are less frequently cited in ethnomedicinal records. However, in South Africa, seeds are brewed to make infusions aimed at treating venereal diseases, infertility, impotence, gonorrhea, and even opportunistic infections linked to HIV [42,43,44]. Though rarely documented, these uses point toward a niche application in reproductive disease management.

Seed use in other *Senna* species is also limited, with reports available for only a few species. In India, *Senna tora* (L.) Roxb. seeds are used to regulate blood lipids, suggesting interest in metabolic health [45]. *S. occidentalis* seeds are also noted in Indian medicine, though details remain vague [46]. This trend indicates that, while the genus is rich in ethnomedicinal uses, seeds are generally underutilized or underinvestigated compared to leaves and roots. This may be due to restricted seasonal availability and preparation challenges.

### 3.5. Stem and Bark

The stem and bark of *S. petersiana* are referenced in scientific literature, but detailed accounts of their traditional medicinal uses are surprisingly limited. This could reflect a true secondary role for these parts in practice, or it may simply highlight gaps in documentation [19]. In contrast, related *Senna* species provide clearer insight into the potential of these parts.

*S. occidentalis* stem is traditionally used in India to aid fracture healing and treat bone disorders, indicating a structural or mineral supporting property [47]. In Mexico, the stem of *Senna racemosa* (Mill.) H.S.Irwin and Barneby is used to manage diabetes, diarrhea, fever, and abdominal pain, common issues in tropical medicine [48]. Similarly, the stem bark of *Senna singueana* (Delile) Lock, used in Kenya and Burkina Faso, is used for the management of malaria and diabetes [49].

These examples suggest that, across the genus, stems and bark play a meaningful role, especially in chronic and infectious diseases. The relative absence of detailed uses for *S. petersiana* may therefore reflect underexploitation rather than irrelevance, considering the success of bark and stem treatments in other species.

**Table 1:** Ethnobotanical uses of different plant parts of *S. petersiana*.

Plant Part Used	Preparation	Ethnobotanical Use	References
Leaves	Decoction; infusion	Malaria, typhoid fever, constipation, intestinal, worn infestation, cough, colds, helminths, syphilis, ethnoveterinary, stomach-ache and intestinal worms	[10,19,21,30]
Roots	Powdered infusion; decoction; burnt to charcoal	Coughs, stomach-aches, constipation, sexually transmitted diseases, malaria, snake bites, intestinal worms and bilharzia (schistosomiasis), ethnoveterinary, fevers, skin infections	[19,20,28,29,30]
Seeds	Powder brewed in water	Venereal diseases, infertility, constipation, gonorrhea impotence, gonorrhea, HIV, and opportunistic infections	[42,43,44]
Pods/Fruits	Eaten raw or cooked	Food-gruel	[10]
bark	No specific records	No specific records	No specific records
Whole plant	Decoction or cold maceration	Convulsions, bone pain, arthritis, diabetes, cholera, fever	[21,22]

## 4. Phytochemical Composition and Quantification of *S. petersiana*

Phytochemical research forms the foundation for understanding the therapeutic potential and biological activities of medicinal plants. By identifying, isolating, and quantifying the active compounds, researchers gain insight into the mechanisms underlying the plants’ traditional uses and their possible pharmacological applications [3]. Over the years, *S. petersiana* has attracted increasing attention due to its broad ethnomedicinal relevance across Africa, prompting efforts to elucidate its complex chemical composition. As summarized in this section, numerous studies have explored the phytochemical profile of *S. petersiana* using diverse extraction solvents and analytical approaches. These investigations have revealed a broad spectrum of bioactive compounds belonging to various chemical classes, emphasizing the plant’s biochemical diversity and its potential as a valuable source of natural therapeutic agents. The phytochemical composition of the plant is explained thoroughly below and in Table 2.

### 4.1. Phytoconstituent Screening

Qualitative analyses of *S. petersiana* have been conducted to determine the presence of various biologically essential phytochemicals. Alkaloids, flavonoids, phenolics, tannins, saponins, phlobatannins, steroids, triterpenes, cardiac glycosides, anthraquinones, and anthocyanins have all been detected in at least one plant part, if not all, underscoring their biological significance [50]. Qualitative phytochemical screening is an essential preliminary step in understanding a plant’s chemical composition, providing insight into which metabolite groups are present and guiding further quantitative investigations. These qualitative findings assist researchers in identifying the classes of compounds that may occur in higher abundance and contribute to the plant’s pharmacological potential.

Quantitative analysis revealed that aqueous extracts of *S. petersiana* leaves contained the highest total phenolic content (1239.94 ± 0.18 mg GAE/g) and relatively low total tannin content (14.14 ± 0.24 mg GAE/g), and alkaloid content (15.76 ± 0.32 mg APE/g) (*p* < 0.05) [51]. These high levels of phenolics likely contribute to the notable biological activities noted in the aqueous extracts. Acetone extracts of *S. petersiana* stems also showed noteworthy amounts of phenolics (269.89 ± 3.05 mg GAE/g) and flavonoid contents (755.87 ± 5.59 mg QE/g) [52]. Interestingly, stems and leaves differ significantly in their polyphenol quantities, suggesting that different plant parts may have different polyphenols to meet specific functions and environmental challenges. Additionally, Laher et al. found that stored leaves contain higher polyphenol levels than fresh leaves, signifying that storage practices; often common in traditional medicine may influence the chemical composition and therapeutic strength of the plant [53]. Olofinsan et al. reported that dichloromethane (DCM) extracts contained significantly higher flavonoid concentrations (*p* < 0.05) compared to other extracts. However, their total phenolic content was lower than that of methanol and aqueous extracts [9]. Moreover, Aremu et al. documented the quantifiable presence of phenolics, gallotannins, condensed tannins, and flavonoids. This confirms the findings that polar solvents generally extract higher amounts of polyphenols than non-polar solvents [54].

This observation aligns with findings from related species within the *Senna* genus. For instance, Gololo et al. evaluated the total phenolic, tannin, flavonoid, and saponin contents of *Senna italica* extracts prepared using hexane, DCM, and methanol. Their results revealed that acetone and methanol extracts contained higher concentrations of these phytochemicals than the non-polar hexane and DCM extracts [55]. Such consistent trends across *Senna* species reinforce the influence of solvent polarity on the extraction efficiency of secondary metabolites, particularly polyphenols and flavonoids.

### 4.2. Identified Compounds of S. petersiana

*S. petersiana* is a chemically diverse species, with a broad spectrum of phytochemicals identified, mostly reported in its leaves, but also in seeds and bark. Its leaves contain amino acids such as hercynine and L-lysine citrate, alongside carbohydrate derivatives including methyl α-D-mannopyranoside, β-D-ribofuranoside methyl, and 2-acetamido-2,3-dideoxy-D-glucose [9,11,51]. These compounds contribute to energy metabolism and cellular functions, supporting traditional uses of leaves and seeds for wound healing and gastrointestinal relief. Polysaccharides such as galactomannan and O-acetyl-glucuronoarabinoxylan have been reported in other *Senna* species like *Senna multijuga* (Rich.) H.S.Irwin and Barneby [56].

Anthraquinones are prominent in *S. petersiana*, including emodin, chrysophanol, rhein, cassiollin, and 4α-acetyl-3,7-dihydroxy-3,6-dimethyldihydronaphthalenone [38,57]. These compounds are known for laxative, antimicrobial, antioxidant, and anti-inflammatory activities, consistent with the plant’s traditional use for gastrointestinal disorders and infections. Similar anthraquinones, such as aloe-emodin, physcion, and floribundone-1, have been identified in *S. alata* and *Senna multiglandulosa* (Jacq.) H.S.Irwin and Barneby [58,59], illustrating common bioactive chemistry across the genus.

The alkaloid-rich extract comprised carbazole and pyrrolidine derivatives such as veratramine, cassine, solasodine acetate, solasodine glucoside, 1,3,6,8-tetra-tert-butyl-9H-carbazole, and 2,3-bis(1-methylallyl)pyrrolidine, alongside other nitrogenous compounds including 3-ethyl-4-methyl-1H-pyrrole-2,5-dione, 6-methylpyridazin-3(2H)-one, and 2,6-dihydroxybenzaldehyde semicarbazone [8,9,11]. A comparative analysis of the alkaloids identified in *S. petersiana* with those reported across the *Senna* genus reveals both structural and biosynthetic similarities. Notably, the presence of cassine directly aligns with previous reports of (-)-cassine, iso-6-cassine, and (-)-3-O-acetylcassine as major alkaloids within the *Senna* genus [60,61]. These compounds share structural features such as piperidine or pyrrolidine nucleus, which may be characteristic of *Senna* alkaloids and contribute to their biological activity.

Chromones have also been characterized [10,28,62]. These metabolites exhibit antimicrobial, antioxidant, and anti-inflammatory effects, supporting the ethnomedicinal application of stems and leaves for infections and inflammatory conditions.

Flavonoids and phenolics in *S. petersiana*, such as quercetin, rhamnetin-3-neohesperidoside, chrysophanol, physcion, dihydrostilbestrol, p-vinylguaiacol, and 2,4-bis(1,1-dimethylethyl) phenol, support antioxidant, antimicrobial, anti-inflammatory, and hepatoprotective activities [9,11,51]. Other *Senna* species, such as *Senna gardneri* (Benth.) H.S.Irwin and Barneby, *Senna georgica* H.S.Irwin and Barneby and *Cassia hirsuta* L. contain flavonoids such as kaempferol, rutin, and dihydromyricetin, as well as phenolics including vanillic and syringic acids [63,64], demonstrating both shared and varying patterns of secondary metabolites.

Terpenoids, including phytol, phytol acetate, β-elemene, α-humulene, β-caryophyllene, α-copaene, squalene, and trans-geranylgeraniol have been reported [9,11]. These compounds contribute to antioxidant, anti-inflammatory, and antimicrobial activities [65]. Many of these terpenoids, such as (E)-phytol, β-caryophyllene, and α-humulene, are widespread in other *Senna* species [63,64], reflecting a conserved chemical framework within the genus.

Sterols and tocopherols, including β-sitosterol and α-tocopherol, are linked to anti-inflammatory, antioxidant, and cholesterol-lowering effects [9,28]. Similar sterols, such as stigmasterol and γ-sitosterol, occur across the genus [66,67]. Fatty acids and long-chain alcohols like methyl palmitate, pentadecanoic acid, hexanoic acid, octadecanoic acid, γ-linolenate, glyceryl-1-hexacosanoate, glyceryl-1-tetracosanoate, n-nonadecanol-1, 1-heptacosanol, and 3,7,11,15-tetramethyl-2-hexadecen-1-ol are also present [9,51] and contribute to antimicrobial and anti-inflammatory properties [68].

Overall, the phytochemical composition of *S. petersiana* shows a combination of widespread metabolites shared across the *Senna* genus and some compounds that are less commonly reported in related species. These constituents collectively support the plant’s ethnomedicinal uses, including treatment of infections, gastrointestinal disorders, and inflammation, while providing a foundation for further pharmacological investigation. 

**Table 2:** Phytochemical compounds identified in different extracts of *S. petersiana*.

Plant Part	Phytochemical Class	Phytochemicals Reported	References
Leaves	Amino acids	Hercynine; L-Lysine citrate	[51]
	Anthracenone	4-Acetyl-3,4-dihydro-3,8-dimethyl-3-hydroxy-6-methoxyanthracen-1(2H)-one	[10]
	Anthocyanidins	Columnidin; 3,3′,4′,5,5′,7-hexahydroxyflavylium	[41]
	Alkaloids	1,3,6,8-Tetratert-butyl-9H-carbazole; 3-Ethyl-4-methyl-1H-pyrrole-2,5-dione; 6-Methylpyridazin-3(2H)-one; 2,3-Bis(1-methylallyl) pyrrolidine; 2,6-Dihydroxybenzaldehyde semicarbazone; veratramine; Cassine; Solasodine glucoside; Solasodine, acetate;	[8,9,11]
	Benzoic acid	5-Methyl-3-(propan-2′-on-1′-yl) benzoic acid; 5-(Methoxymethyl)-3-(propan-2′-ol-1′-yl) benzoic acid	[62]
	Carbohydrates	Methyl α-D-mannopyranoside; Beta-D-ribofuranoside, methyl; 2-Methyl-D-glucose	[9]
	Chromones	7-Acetonyl-5-hydroxy-2-methylchromone; 7-(Propan-2′-ol-1′-yl)-5-hydroxy-2-methylchromone; 5-Acetonyl-7-hydroxy-2-hydroxymethylenchromone; 5-Acetonyl-7-hydroxy-2-methylchromone; 4a,7,7,10a-Tetramethyldodecahydrobenzo[f]chromen-3-ol	[10,11,28,62]
	Dihydroanthracenone	4α-Acetyl-3,7-dihydroxy-3,6-dimethyldihydronaphthal-enone	[28]
	Fatty acids	Methyl palmitate; Pentadecanoic acid, Hexanoic acid; Octadecanoic acid; glyceryl-1-hexacosanoate, gamma-linolenate; Glyceryl-1-tetracosanoate;	[9,28,51,62]
	Fatty alcohols	n-Nonadecanol-1; 1-Heptacosanol; 3,7,11,15-Tetramethyl-2-hexadecen-1-ol	[9,11]
	Flavonoids	Quercetin; rhamnetin-3-neohesperidoside; chrysophanol; physcion;	[9,11,51]
	Sugars and Sugar Derivatives	2,4:3,5-Dimethylene-L-iditol—Polyol (sugar alcohol); 2-Acetamido-2,3-dideoxy-D-glucose; Beta-D-ribofuranoside, methyl; 2-Methyl-D-glucose	[11]
	Phenolics	Dihydrostilbestrol; p-Vinylguaiacol; Phenol,2,4-bis(1,1-dimethylethyl);	[9,11]
	Terpenoids/Sterols	Phytol; Phytol acetate; Phytol, acetate; β-sitosterol; β-elemene; α-humulene; β-caryophyllene; α-copaene; Pytol; Squalene; trans-Geranylgeraniol; α-Tocopherol; Sitosterol-3-O-β-D-glucoside, Stigmasterol-3-O-β-D-glucoside	[9,11,28,50]
	Terpenes	Dihydroactinidiolide	[9]
bark	Flavonoids	Cassiaflavan dimers and trimers (proanthocyanidins)	[29]

### 4.3. Isolated Compounds

*S. petersiana* produces a diverse array of isolated secondary metabolites that reflect both the common chemical framework of the *Senna* genus and species-specific traits. Most isolates have been reported from the leaves and bark, though compounds have also been obtained from the seeds.

**Figure 1:**
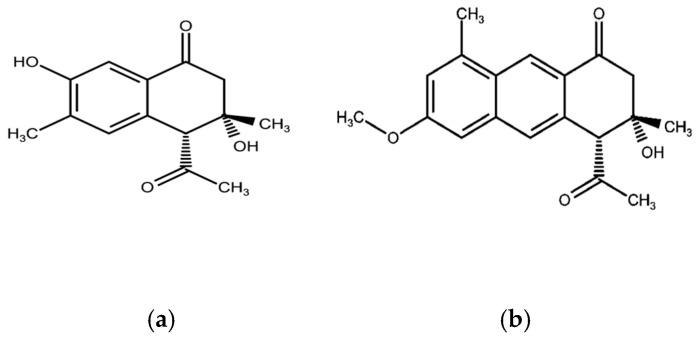
Structures of (**a**) dihydronaphthalenone (4α-Acetyl-3,7-dihydroxy-3,6-dimethyldihydronaphthalenone) [10] and (**b**) anthracenone (4-Acetyl-3,4-dihydro-3,8-dimethyl-3-hydroxy-6-methoxyanthracen-1(2H)-one) [54] isolated from *S. petersiana*.

**Figure 2:**
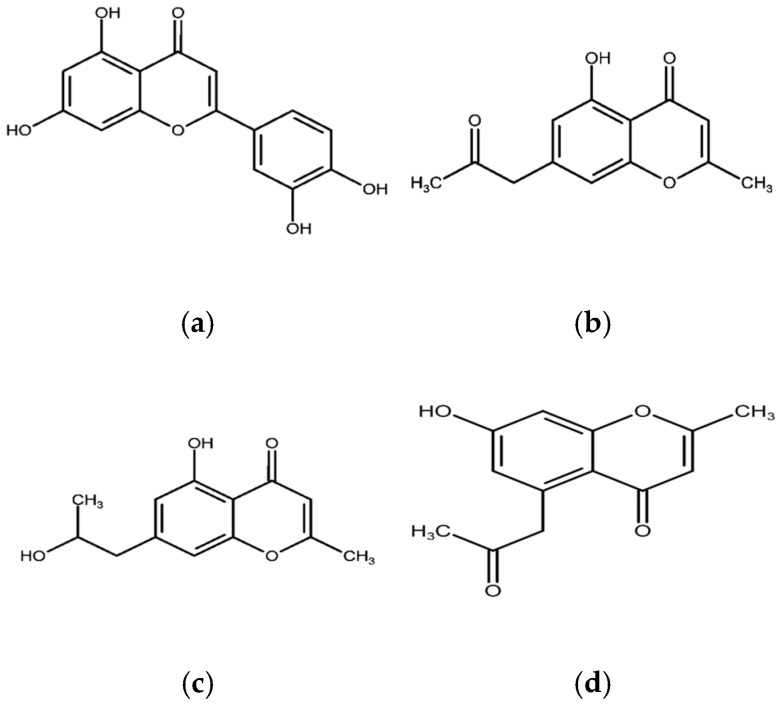
Structures of chromones namely (**a**) 5-Acetonyl-7-hydroxy-2-hydroxymethylenchromone [54], (**b**) 7-Acetonyl-5-hydroxy-2-methylchromone [25,62], (**c**) 5-Hydroxyl-2-methyl-7-(propan-2β-ol)-chromone [25] and (**d**) 5-Acetonyl-7-hydroxy-2-methylchromone [54] isolated from *S. petersiana*.

Among the key isolates are anthracenone and dihydroanthracenone derivatives such as 4-acetyl-3,7-dihydroxy-3,6-dimethyldihydronaphthalenone (Figure 1a) and 4-acetyl-3,4-dihydro-3,8-dimethyl-3-hydroxy-6-methoxyanthracen-1(2H)-one (Figure 1b) [10,57]. These compounds are biosynthetically related to the anthraquinones commonly found in other *Senna* species, such as emodin, which has been shown to exhibit laxative activity and promote wound healing [68,69]. Given that *S. petersiana* is traditionally used as a laxative, its pharmacological effect may be partly attributed to the presence of these anthracenone and dihydroanthracenone derivatives. This relationship suggests that *S. petersiana* follows the same polyketide biosynthetic pathway typical of the genus but tends to produce reduced anthracenones rather than oxidized anthraquinones. Such variation may represent a metabolic specialization that distinguishes *S. petersiana* from its close relatives while preserving the biological functions associated with this class of compounds [70]. Chromones form another dominant chemical group in *S. petersiana*. In addition to the identified ones, several acetonyl-substituted hydroxychromones have been isolated (Figure 2), including (a) 5-Acetonyl-7-hydroxy-2-hydroxymethylenchromone [57], (b) 7-Acetonyl-5-hydroxy-2-methylchromone [28,62], (c) 5-Hydroxyl-2-methyl-7-(propan-2β-ol)-chromone [28] and (d) 5-Acetonyl-7-hydroxy-2-methylchromone (e) [57]. Some of these metabolites, commonly referred to as petersinones, showed antioxidant activity even though it was weak [28]. Chromones such as 5-acetonyl-7-hydroxy-2-methylchromone have also been reported in other *Senna* species, such as *S. siamia* [66]; however, the petersinone series appears to be predominant in *S. petersiana*. Their repeated characterization and isolation from different plant parts and across multiple studies suggest that these chromones could serve as stable chemotaxonomic markers for the species.

**Figure 3:**
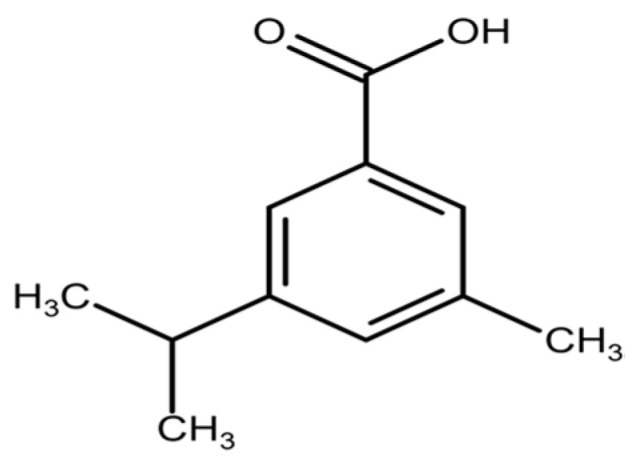
Structure of benzoic acid derivative (5-Methyl-3-(propan-2-on-1-yl) benzoic acid [62].

In addition, benzoic acid derivatives such as 5-methyl-3-(propan-2-on-1-yl) benzoic acid (Figure 3) and 5-(methoxymethyl)-3-(propan-2-ol-1-yl) benzoic acid have been reported [28]. Structurally related benzoic acids are found in *S. occidentalis*, where they contribute to the antioxidant activity of the plant [71].

**Figure 4:**
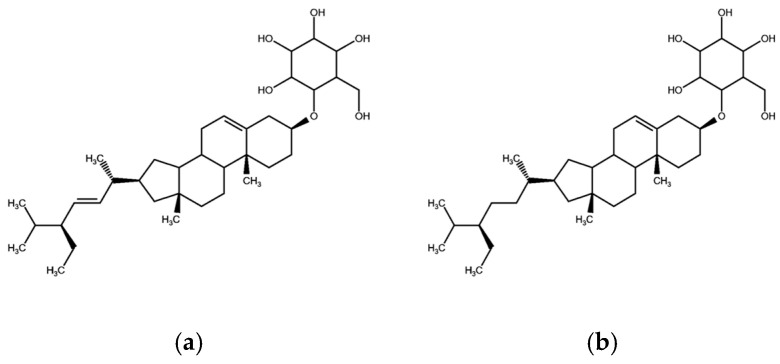
Structures of sterol glycosides namely (**a**) Stigmasterol-3-O-β-D-glucoside [10,25,54] and (**b**) β-Sitosterol-3-O-β-D-glucoside [10,59] isolated from *S. petersiana*.

**Figure 5:**
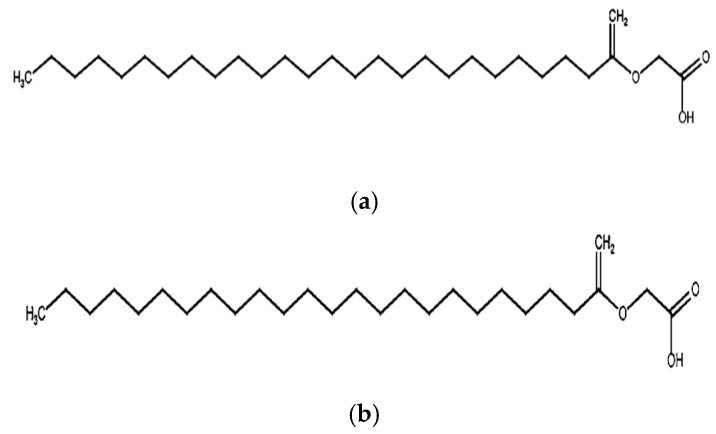
Structures of fatty acid esters namely (**a**) Glyceryl-1-hexacosanoate [25] and (**b**) Glyceryl-1-tetracosanoate [62] isolated from *S. petersiana*.

Sterol glycosides and fatty acid derivatives are also well represented in *S. petersiana*. The sterols stigmasterol-3-O-β-D-glucoside (Figure 4a) and β-sitosterol-3-O-β-D-glucoside (Figure 4b) have consistently been isolated [10,28,57]. Stigmasterol and β-sitosterol and are widely distributed across *Senna* species, including *Senna sieberiana* (DC. ex Collad.) H.S.Irwin and Barneby and *S. siamea* [72,73], and are known for their anti-inflammatory and membrane-stabilizing effects [74,75]. Similarly, long-chain fatty acid esters such as glyceryl-1-tetracosanoate and glyceryl-1-hexacosanoate (Figure 5a,b) have been isolated from the plant. The presence of high-mass glyceryl derivatives in *S. petersiana* may enhance lipid solubility and facilitate interaction with biological membranes, which could support the plant’s use in treating inflammatory and infectious conditions [76,77].

**Figure 6:**
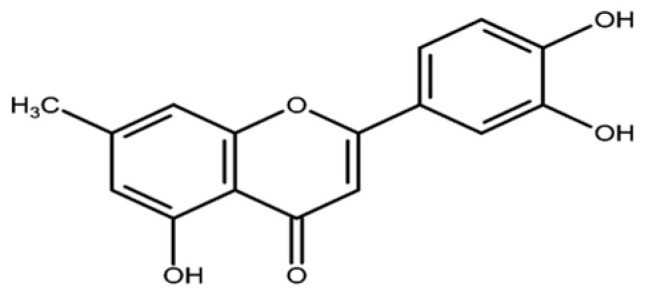
Structures of flavonoid (Luteolin) [80] isolated from *S. petersiana*.

Flavonoids, although less abundant, play an important role in the chemistry of *S. petersiana*. Luteolin has been isolated from the seeds of *S. petersiana* (Figure 6) [78]. This flavonoid has also been isolated from other *Senna* species such as *S. singueana, S. siame* and *S. alata* [79,80,81]. The compound has been reported to contribute to antiulcer, antibacterial, strong antioxidant and anti-lipoxygenase activity in *S. singueana* [79,82]. In *S. petersiana*, luteolin appears to be one of the predominant flavonoid constituents, whereas quercetin and kaempferol derivatives are more common in other *Senna* species [79,80,81].

*S. petersiana* exhibits both shared and unique chemical features compared to other members of the genus. The consistent occurrence of anthracenones, chromones, sterol glycosides, and fatty acid esters highlights its close biochemical relationship with other *Senna* species. In contrast, the acetonyl-substituted chromones and methylated benzoic acids appear more specific to this species. These findings not only support its traditional medicinal applications for infections and inflammation but also position *S. petersiana* as a chemically distinct member of the *Senna* genus, warranting deeper pharmacological exploration.

## 5. Biological Activities of *S. petersiana*

The biological activities of *S. petersiana*, explored below and further highlighted in the Table 3, offer valuable insights. Many of the reported effects, such as antimicrobial, anti-inflammatory, and antiparasitic properties, closely align with the way it has been used for generations to treat infections, digestive issues, and inflammatory conditions. These findings not only support traditional knowledge but also highlight the plant’s potential for future pharmacological research.

### 5.1. Antibacterial Activity

*S. petersiana* exhibits potent antibacterial activity, supporting its traditional use in treating various bacterial infections, including those caused by multidrug-resistant ESKAPE pathogens such as *Staphylococcus aureus* and *Pseudomonas aeruginosa*. These bacteria are common causes of hospital-acquired infections and are resistant to major antibiotics, such as β-lactams, carbapenems, and tetracyclines [83].

Several in vitro investigations using agar well diffusion, TLC bioautography, and microbroth dilution assays have provided both qualitative and quantitative evidence of S. *petersiana* antibacterial potential. Notably, Tshikalange et al. demonstrated that ethanol extracts of seeds inhibited Gram-positive bacteria (*Bacillus cereus*, *Bacillus pumilus*, *Bacillus subtilis*, *S. aureus*) and Gram-negative strains (*Enterobacter cloacae* and *Serratia marcescens*), with MIC values of 20 mg/mL. Surprisingly, TLC bioautography revealed only a single inhibition band against *S. aureus*, underscoring the greater sensitivity and reliability of quantitative MIC assays compared with qualitative assessments. Further isolation and testing of luteolin, a flavonoid compound from the seeds, revealed significantly enhanced antibacterial activity at 1 mg/mL against the same Gram-positive strains, aligning with a growing body of literature emphasizing flavonoids’ critical role in phytochemical-driven antimicrobial therapies [80,84,85].

Broad-spectrum antibacterial effects were also reported for methanol and acetone extracts of leaves and stems, showing potent inhibition of *S. aureus*, *Escherichia coli*, *Enterococcus faecalis*, *P. aeruginosa*, and *Klebsiella pneumoniae*, with MIC and MBC values ranging from 0.08 to 0.63 mg/mL [51,52]. Laher et al. similarly documented MICs below 1 mg/mL for ethanol extracts against *S. aureus* and *E. coli*, confirming strong antibacterial potency [53]. Aremu et al. classified MIC values under 1 mg/mL as effective, and *S. petersiana* ethanol extracts met this threshold with MICs of 0.39 mg/mL for *B. subtilis*, *S. aureus*, and *K. pneumoniae*, as well as moderate activity against *E. coli* (0.78 mg/mL) [54]. This non-selective activity suggests bioactive compounds target conserved bacterial structures common to both Gram-positive and Gram-negative bacteria.

When evaluated against mycobacteria, *S. petersiana* showed variable activity. Extracts displayed moderate inhibition of *Mycobacterium smegmatis* (MIC 0.63–2.5 mg/mL) but lacked activity against *Mycobacterium tuberculosis*, underscoring the limitations of surrogate models and the need for direct testing against pathogenic strains [51]. Similarly, Nyambo et al. reported weak antimycobacterial effects for most crude extracts, except a dichloromethane (DCM) extract that potently inhibited *Mycobacterium aurum* (MIC 0.04 mg/mL) [86].

The antibacterial effects of *S. petersiana* extend to clinically significant *Salmonella* species. DCM: methanol leaf extracts exhibited inhibition zones of 14–18 mm and MIC/MBC values of 1.5 mg/mL and 12 mg/mL, respectively, against *Salmonella typhi*, *Salmonella paratyphi A*, and *Salmonella paratyphi*. However, isolated chromone derivatives from the same extracts lacked activity, highlighting the complexity of synergistic effects in crude extracts [28]. Comparative studies revealed that leaves demonstrated the highest antibacterial efficacy (zones 13–19 mm) compared to flowers, roots, and bark. Leaf extract was further evaluated using a broth microdilution assay, where it had MIC and MBC values of 1 mg/mL and 8 mg/mL, respectively, affirming the plant’s antimicrobial activity [50]. The antibacterial effectiveness of *Senna* species varies widely, as *Senna alata* (L.) Roxb. showed good inhibition against *Salmonella typhimurium* (zones 10–12 mm) [87], whereas *S. siamea* exhibited poor activity (zones 1–10 mm) [88].

Isolation of stigmasterol-3-O-β-D-glucoside from DCM: methanol leaf extracts revealed superior anti-*Salmonella* activity, with inhibition zones between 15 and 21 mm and MIC/MBC values of 22.5 µg/mL and 90 µg/mL, respectively, outperforming the crude extract. This underscores the importance of isolating pure bioactive compounds to remove inactive or antagonistic constituents and to facilitate mechanistic studies and drug development. With MIC values below 0.1 mg/mL typically considered potent, *S. petersiana*’s pure compounds show promising pharmacological potential [57].

Despite the roots being the most commonly used part in traditional medicine, there remains a notable gap in scientific evaluation of their antibacterial properties. Bridging this gap will be crucial to validate ethnobotanical knowledge and may lead to the discovery of novel antibacterial agents. The consistent antibacterial activity of *S. petersiana* against pathogens responsible for urinary tract, skin, and gastrointestinal infections validates its traditional medicinal applications. Furthermore, the genus *Senna* exhibits diverse but generally broad-spectrum antimicrobial effects, highlighting the ethnopharmacological and therapeutic significance of these species as promising sources of natural antibacterial compounds.

### 5.2. Anti-Virulence Activities

Bacterial virulence factors, such as biofilm production, motility, toxin production, adhesion, and quorum sensing, are essential for pathogenicity, facilitating activities such as colonization and invasion of host tissues [89]. These mechanisms not only promote bacterial survival and transmission but also facilitate chronic and treatment-resistant infections [90]. In contrast to traditional antibiotics, which act by killing bacteria, anti-virulence approaches aim to disarm virulence mechanisms without exerting bactericidal pressure, thereby diminishing the emergence of resistance [91]. Phytochemicals, including flavonoids, terpenes, and phenolic acids, can interfere with biofilm formation, inhibit motility, and disrupt quorum-sensing pathways, and have been described as potential candidates for anti-virulence therapy [92].

Even though several plant species have been studied for this property, research on *S. petersiana* is limited. To date, only its anti-biofilm and anti-motility abilities have been tested but still lacking, and no information has been reported regarding its ability to decelerate the production of other virulent factors, such as quorum sensing, protease secretion, and toxin production. Crude extracts of *S. petersiana* demonstrated low potency in anti-virulence assays. CV staining showed an initial adherence inhibition rate of less than 50% in *P. aeruginosa*, *K. pneumoniae*, *E. coli*, *S. aureus*, and *E. faecalis*, and an increase in biofilm biomass in the aforementioned Gram-positive strains. Likewise, anti-swarming bioassays at sub-inhibitory concentrations resulted in less than 50% inhibition of motility in *P. aeruginosa* and *E. coli* [52].

However, other species of the *Senna* genus have demonstrated significant anti-virulence activity. For example, the stem bark extract of *S. siamea* showed impressive biofilm-inhibitory activity against *E. coli* [93], and *Cassia fistula* L. demonstrated vigorous antibiofilm activity against *S. aureus* [94]. Moreover, acetone and hexane extracts of *S. alexandrina* effectively inhibited quorum-sensing-controlled virulence profiles mediated by *P. aeruginosa*, including pyocyanin (75%) and biofilm (62%) formation, as well as swarming and protease activity [95]. These findings illustrate the wider anti-virulence potential in the genus.

Although crude extracts of *S. petersiana* display poor anti-virulence potential, the broad spectrum of bioactive secondary metabolites of the genus and the reported efficacy of related species justify further studies. Further work on bioassay-guided fractionation to isolate the responsible constituents and on elucidation of their effects on quorum sensing and other virulent pathways is required. The evaluation of in silico strategies, including molecular docking, could also help track promising anti-virulence entities. Furthermore, examining the synergistic actions of plant-derived compounds combined with antibiotics may provide new resources to effectively fight multidrug-resistant pathogens.

### 5.3. Antioxidant Activity

Oxidative stress, primarily driven by reactive oxygen species, is now recognized as a central factor in the onset and progression of several chronic illnesses such as cardiovascular disease, diabetes, neurodegenerative disorder and inflammatory conditions [96]. Plants have a variety of compounds that counter this damage by neutralizing radicals or boosting the body’s own antioxidant system [97]. To evaluate the effects of phytochemical compounds from *S. petersiana* on these radicals, assays such as the DPPH radical scavenging assay, ferric reducing power assay, and hydrogen peroxide antioxidant assay have been used. Potency is usually expressed as % inhibition, EC_50_, or IC_50_ values.

The antioxidant activity of *S. petersiana* has been reported across a range of solvents. Matotoka et al. showed that the aqueous leaf extract had only moderate efficacy, with EC_50_ values of 272 µg/mL (DPPH) and 178 µg/mL (FRAP). The other extracts tested in the study (hexane, dichloromethane, acetone, and methanol) were generally weak, with effective concentrations above 500 µg/mL [51]. However, the acetone and methanol stem extracts were highly active, with DPPH EC_50_ values of 17.73 and 18.09 µg/mL, respectively. The acetone stem extract also showed strong reducing power, with an EC_50_ of 14.57 µg/mL, which was even better than that of ascorbic acid (48.42 µg/mL) [52].

Olofinsan et al. also reported supporting findings, with the dichloromethane extract showing an IC_50_ of 52 µg/mL in the FRAP assay. They also evaluated the effect of plant extracts on hydroxyl radicals. All their extracts scavenged hydroxyl radicals (DCM, methanol, and distilled water), with the DCM fraction (IC_50_ = 44.7 µg/mL) almost matching that of gallic acid (44.9 µg/mL). The aqueous extract also showed a dose-dependent effect, with an IC_50_ of 37.3 µg/mL, though not as strong as ascorbic acid (23.2 µg/mL) [9]. Looking across these reports, crude extracts of *S. petersiana* often land below the 100 µg/mL threshold in IC_50_/EC_50_ terms and therefore can be described as having a generally moderate antioxidant capacity [98]. An interesting observation came from Laher et al., who evaluated the effects of stored leaves compared with fresh leaves and found that stored leaves were better antioxidants than fresh ones. The EC_50_ value for short-term storage was 0.63 µg/mL, compared to 0.82 µg/mL for fresh leaves, suggesting that post-harvest biochemical shifts, or perhaps the degradation of inhibitory compounds, might enhance activity. It is an odd but not implausible finding, hinting that timing and handling matter as much as solvent choice [53].

Isolated compounds were also evaluated for antioxidant activities. Most of the pure constituents tested showed weaker radical-scavenging activity than the crude mixtures they were derived from, suggesting some level of synergy within the extract. Four compounds namely, 7-acetonyl-5-hydroxy-2-methylchromone, 5-hydroxy-2-methyl-7-(propan-2β-ol)-chromone, glyceryl-1-hexacosanoate, and stigmastosterol-3-O-β-D-glucoside were evaluated for antioxidant activity. Only the stigmastosterol-3-O-β-D-glucoside had promising effects, with a scavenging activity (SC_50_) of 36 µM. The other compounds showed no noteworthy activity [28].

Compared with other *Senna* species, *S. petersiana* has demonstrated antioxidant activity similar to that of other *Senna* species when evaluated using comparable assays. *S. siamea* methanol extract showed notable antioxidant effects, recording IC_50_ values of 20.63 µg/mL and 11.05 µg/mL against DPPH and ABTS (3-ethylbenzothiazoline-6-sulfonate) radicals, respectively [99]. However, not all *Senna* species exhibit equivalent potency. For example, *S. alata* methanolic and aqueous extracts showed markedly weaker DPPH scavenging effects, with IC_50_ values of 662 µg/mL and 879 µg/mL, respectively [100]. The antioxidant properties observed across *Senna* species are consistently attributed to high concentrations of phenolics, flavonoids, and anthraquinones [101]; phytochemical groups are also abundant in *S. petersiana*. This consistency indicates that *S. petersiana* shares similar antioxidant compounds with other *Senna* species, supporting its strong antioxidant potential and importance as a natural source of antioxidants.

### 5.4. Anti-Inflammatory Activity

Inflammation involves a web of processes, such as protein denaturation and enzyme activation, that together result in pain, swelling, and tissue damage [102]. Plants have long been tapped as sources of relief, as they contain phytochemicals such as phenolics, flavonoids, and alkaloids that can interfere with inflammatory pathways, sometimes achieving effects similar to synthetic drugs but without the baggage of severe side effects [103]. In vitro, researchers usually probe this activity through protein denaturation assays (Bovine serum albumin or egg albumin heating models are standard) or by testing for enzyme inhibition, most often cyclooxygenases (COX-1 and COX-2) [104].

For *S. petersiana*, though the literature on this aspect is limited, what has been evaluated so far looks promising. Matotoka et al. showed that aqueous and methanol leaf extracts strongly suppressed heat-induced BSA denaturation, outpacing the hexane fraction. The aqueous extract also curbed egg albumin denaturation by more than 60% [51]. In a different study, Aremu et al. reported almost complete inhibition of COX-1 (99.3 ± 1.2%) by the ethanol leaf extract. The other solvent extracts (petroleum ether, DCM, and water) showed noteworthy activity, with percentage inhibitions greater than 70%. In contrast, the same ethanol leaf extract showed 47.8 ± 3.8% inhibition of COX-2, while the other solvents showed inhibitions ranging from 56.8% to 65.2% [54].

These findings indicate selective modulation of inflammatory enzymes, supporting the plant’s potential as a source of bioactive compounds with anti-inflammatory properties. Notably, such activity closely corresponds to traditional uses of *S. petersiana* for the treatment of bone pain, arthritis, and fever, conditions commonly associated with inflammatory processes [20]. The extracts’ ability to suppress protein denaturation and COX enzyme activity provides pharmacological support for these ethnomedicinal claims, reinforcing the traditional use of leaf and whole plant preparations for managing inflammatory ailments.

### 5.5. Antiviral and Anthelmintic Activity

Research on the antiviral potential of *S. petersiana* is still sparse, with only a handful of early in vitro studies. Tshikalange et al. tested both luteolin, a flavonoid isolated from the plant, and crude seed extracts using a cytopathic effect (CPE) assay. At non-toxic concentrations (≤250 µg/mL), luteolin reduced viral CPE by about 50%. The crude seed extracts performed somewhat better at certain doses, showing 30% reduction at 31 µg/mL and 65% reduction at 125 µg/mL. Even so, the authors cautioned that much of this antiviral activity might be a side effect of general cytotoxicity, rather than a true selective antiviral effect [79].

Similarly to the anthelmintic properties of *S. petersiana,* research on its activity is still quite limited, with only a few studies reporting its activity. In one investigation, Aremu et al. tested extracts prepared using petroleum ether, dichloromethane, ethanol, and water against *Caenorhabditis elegans*. They categorized minimum lethal concentration (MLC) values below 1 mg/mL as showing high activity, between 1 and 4 mg/mL as moderate, and above 4 mg/mL as low. The ethanol extract gave the strongest effect, with an MLC of 0.52 mg/mL, while petroleum ether and dichloromethane extracts both showed moderate activity at 1.04 mg/mL. The water extract was much weaker, with an MLC of 8.33 mg/mL [54].

Although the results of both antiviral and anthelmintic activities indicate promising efficacy, there is still a clear need for more detailed work, as published data on this species remain scarce. Given that flavonoids and anthraquinones from related *Senna* species are already known to have antiviral activity, these early results are intriguing but primarily highlight a significant research gap [5]. Selectivity indices, mechanistic assays, perhaps even animal models, are needed before any conclusions can be drawn about the real antiviral and anthelmintic relevance of *S. petersiana*.

### 5.6. Anti-Tumour Activity

Cancer remains a leading cause of mortality worldwide, and the search for safer, plant-derived anticancer agents is ongoing [105]. Phytochemicals have shown anti-proliferative effects across various models, making plants such as *S. petersiana* worth closer examination.

Evidence from several studies suggests that *S. petersiana* has activity against breast cancer cell lines. Olofinsan et al. tested both chloroform and ethyl acetate leaf extract rich in alkaloids against MCF-7 breast cancer cells. The ethyl acetate fraction reduced cell viability to 43.9% in standard MTT assays, whereas the chloroform extract left 66.3% of cells viable, indicating the ethyl acetate fraction is more potent. When combined with laser light, both extracts became even more cytotoxic, suggesting the presence of photoreactive compounds that may act as photosensitizers, a property relevant to photodynamic therapy [8].

Other work by Nyambo et al. examined triple-negative breast cancer (MDA-MB-231 cells) and found that intermediate-polarity extracts, particularly chloroform and dichloromethane (DCM), exerted strong anti-proliferative effects. At the lowest concentration tested (62.5 µg/mL), both extracts inhibited viability by more than 50%. The DCM extract was especially potent, with an IC_50_ of 1.53 ± 0.46 µg/Ml; outperforming cisplatin (IC_50_ = 2.02 ± 0.09 µg/mL). The chloroform extract was also active, with an IC_50_ of 26.26 ± 2.33 µg/mL. Interestingly, when tested in HepG2-derived C3A hepatocellular carcinoma cells, no toxicity was observed at concentrations up to 200 µg/mL, suggesting some selectivity [86].

Beyond crude extracts, Djemgou et al. assessed four isolated compounds against several cancer cell lines (HepG2, MCF-7, and 1301 leukemia). Three showed only modest activity at high concentrations, while one compound displayed notable potency against solid tumours (IC_50_ = 82.7 µM). Against MCF-7 cells, IC_50_ values ranged from 68.1 to 143.7 µM, while activity against leukemia cells was minimal, hinting that the cytotoxic effects may be somewhat selective for solid tumours [28].

These findings show that *S. petersiana* holds real promise in cancer research, especially for aggressive breast cancer subtypes. The extracts’ strong performance, even outperforming cisplatin in some assays, and the added photodynamic potential, suggest novel therapeutic applications.

### 5.7. Toxicological Profile

Assessing cytotoxicity is a necessary step in evaluating medicinal plants, not only to assess therapeutic potential but also to flag potential safety concerns. Sometimes, natural compounds kill cancer cells selectively while sparing normal cells, an ideal outcome in drug development [106]. But often, they exert broad toxicity, and this distinction needs to be carefully clarified through systematic testing [107].

In one of the earliest reports, *S. petersiana* extracts showed significant toxicity toward vero monkey kidney (VK) cells, with an ID_50_ of 24 µg/mL. Luteolin, however, appeared relatively safe, as more than two-thirds of VK cells remained viable at 500 µg/mL, and no structural changes were observed at doses below 250 µg/mL [83].

Later studies provided a more detailed understanding of the toxicological effects. Matotoka et al. tested the extracts on human THP-1 macrophages and Vero monkey kidney cells. At 100 µg/mL, no significant effects on THP-1 viability were observed (*p* > 0.05), but cytotoxicity was detected in Vero cells (*p* < 0.05), suggesting that toxicity may depend on the cell model or even the species of origin [51]. Brine shrimp lethality assays, often used as a crude toxicity screen, reported an LC_50_ of 174.3 ± 0.9 µg/mL [52], consistent with moderate toxicity. Interestingly, Nyambo et al. observed no harmful effects on Vero cells at concentrations of 50–200 µg/mL [86].

Comparable results have been reported in other *Senna* species. For instance, extracts of *Senna cana* (Nees and Mart.) H.S.Irwin and Barneby and *Senna pendula* (Willd.) H.S.Irwin and Barneby exhibited low to no toxicity against *Artemia salina* (brine shrimp), with LC_50_ values exceeding 700 µg/mL [108]. Similarly, ethanolic extracts and fractions of *S. macranthera* flowers showed low cytotoxicity on Vero cells (ATCC CCL 81) (between 5.9 and 23.4 µg/mL) [109]. These comparisons suggest that *S. petersiana* may have slightly greater cytotoxicity than some plants in the same genus, particularly in certain cell lines. This could reflect differences in the concentration or type of bioactive compounds in the plants. Nonetheless, the overall in vitro toxicity profile remains within a moderate and manageable range, supporting its continued pharmacological exploration. The observed variability further underscores the need for standardized extraction protocols and cross-species toxicity benchmarks to better contextualize *S. petersiana*’s safety and therapeutic window.

In vivo studies add another layer to understanding the toxicological profile of *S. petersiana*. Acute toxicity tests in mice revealed high LD_50_ values of 20 g/kg for males and 22 g/kg for females, indicating low acute toxicity even at very high single doses. Sub-chronic studies in rats presented a more complex picture: moderate doses (around 75 mg/kg) were well tolerated, whereas higher doses (≥300 mg/kg) resulted in altered serum and urinary protein levels and increased liver and kidney organ weights. At extreme doses (4800 mg/kg), male rats exhibited signs of renal stress, including elevated urinary protein [50]. In comparison, administration of ethanolic and aqueous leaf extracts of *Senna italica* Mill. at 5000 mg/kg in male Wistar rats produced no mortality, morbidity, or behavioural abnormalities, suggesting an absence of acute toxicity for this related species. The absence of toxicity in *S. italica* supports the general safety of *Senna* species and implies that *S. petersiana* may also be relatively safe at therapeutic levels [110]. However, mild renal alterations observed at higher doses in *S. petersiana* suggest possible dose-dependent sensitivities, underscoring the need for careful dose optimization and chronic toxicity assessments to fully establish its safety profile.

The toxicological profile of *S. petersiana* is mixed, in which some extracts appear relatively safe, while others exhibit dose-dependent toxicity. This aligns with the careful preparation methods of traditional medicine, where decoctions and diluted infusions are typically used rather than concentrated extracts. The long-standing ethnomedicinal use of *S. petersiana* for internal and external treatments without widespread reports of harm suggests that traditional dosing practices may have intuitively balanced efficacy with safety. Nevertheless, systematic toxicity profiling and dosage standardization remain essential before clinical or pharmaceutical application.

**Table 3:** Biological activities of *S. petersiana*.

Activity	Plant Part and Extract	Test Organisms/Models	Assay Type/Method	Results	References
Antibacterial	Seeds (ethanol extract)	*Bacillus cereus*, *B. pumilus*, *B. subtilis*, *S. aureus*	Agar diffusion, microbroth dilution, TLC bioautography	MIC-20 mg/mL; TLC showed 1 inhibition band against *S. aureus*	[79,84,85]
	Leaves and stems (acetone and methanol extracts)	*S. aureus*, *E. coli*, *E. faecalis*, *P. aeruginosa*, *K. pneumoniae*	Microbroth dilution	MIC/MBC range: 0.08–0.63 mg/mL	[51,52]
	Leaves, ethanol extract	*S. aureus*, *E. coli*	Microbroth dilution	MIC < 1 mg/mL	[53]
	Leaves (hexane, DCM, acetone, etc.)	*Mycobacterium smegmatis* and *M. tuberculosis*	Microbroth dilution	No activity against *M. tuberculosis*; moderate activity against *M. smegmatis* (0.63–2.5 mg/mL); noteworthy DCM active against *M. aurum* A+ (0.04 mg/mL)	[51,85]
	Leaves (DCM: methanol extract)	*Salmonella typhi*, *S. paratyphi A* and *B*	Agar diffusion, Microbroth dilution	DCM:methanol: inhibition zones 14–18 mm; MIC 1.5 mg/mL; MBC 12 mg/mL	[10,50]
	Leaves, stigmasterol-3-O-β-D-glucoside	Various bacteria	Agar diffusion, Microbroth dilution	Inhibition zones 15–21 mm; MIC 22.5 μg/mL; MBC 90 μg/mL-better than crude extract	[10]
Anti-virulence	Crude extracts	*P. aeruginosa*, *K. pneumoniae*, *E. coli*, *S. aureus*, *E. faecalis*	Biofilm inhibition (crystal violet staining), anti-swarming motility assay	<50% inhibition of biofilm adherence; increased biofilm in Gram-positives; <50% inhibition of motility at sub-MIC	[52]
Antioxidant	Leaves, aqueous extract	DPPH, FRAP assays	EC_50_ values	DPPH EC_50_: 271.77 µg/mL; FRAP EC_50_: 178.23 µg/mL	[51]
	Stem, acetone and methanol extracts	DPPH, FRAP assays	EC_50_ values	DPPH EC_50_: 17.73 and 18.09 µg/mL; FRAP EC_50_: 14.57 µg/mL (better than ascorbic acid at 48.42 µg/mL)	[52]
	DCM extract	Hydroxyl radical scavenging	IC_50_ values	IC_50_: 44.70 µg/mL (similar to gallic acid 44.90 µg/mL); DPPH IC_50_: 51.60 µg/mL, weaker than ascorbic acid	[9]
	Isolated compounds	DPPH assay	SC_50_ values	Only stigmastosterol-3-O-β-D-glucoside is active (SC_50_ = 36 μM), comparable to ascorbic acid	[28]
Anti-inflammatory	Leaves aqueous and methanol extracts	Heat-induced protein denaturation; COX inhibition	BSA, egg albumin denaturation; COX 1 and 2 assay	>60% inhibition of egg albumin denaturation; COX 1 inhibition 99.3 ± 1.2%	[51,54]
Antiviral	Seeds, ethanol extract; luteolin isolated	Viral cytopathic effect assay	CPE reduction assay	Luteolin reduced viral CPE by 50% at ≤250 µg/mL; crude extract 65% reduction at 125 µg/mL; effects possibly due to cytotoxicity	[79]
Anthelmintic	petroleum ether, dichloromethane, ethanol, and water extracts	*Caenorhabditis elegans*	in vitro colourimetric assay	The ethanol extract showed the most potent effect, with an MLC of 0.52 mg/mL, while the petroleum ether and dichloromethane extracts showed moderate activity at 1.04 mg/mL. The water extract was much weaker, with an MLC of 8.33 mg/mL.	[54]
Anti-tumour	Leaves, alkaloid-rich chloroform and ethyl acetate extracts	MCF-7 breast cancer cells	MTT assay; photodynamic therapy	Ethyl acetate extract viability 43.9%; chloroform 66.3%; enhanced reduction with light exposure (~60% viability reduction)	[8]
	Crude extracts (chloroform, DCM)	MDA-MB-231 triple-negative breast cancer cells	Viability assay	>50% viability inhibition at 62.5 µg/mL; DCM IC_50_ = 1.53 ± 0.46 µg/mL (better than cisplatin 2.02 µg/mL)	[86]
	Isolated compounds (4 types)	HepG2, MCF-7, 1301 leukemia cells	Cytotoxicity assays	Compound 4 most potent, IC_50_ 82.7 µM (solid tumour); MCF-7 IC_50_s 68.1–143.7 µM; minimal effect on leukemia cells	[28]
Toxicological Profile	Various extracts	Vero cells, THP-1 macrophages, and the brine shrimp lethality assay	Cytotoxicity assays	VK cells ID_50_ 24 µg/mL; luteolin safe up to 250 µg/mL; THP-1 viability unaffected at 100 µg/mL; Vero cells variable toxicity; brine shrimp LC_50_ 174.3 µg/mL	[51,52,84,86]

## 6. Conclusions

*S. petersiana* contains a diverse range of phytochemicals that contribute to its reported antibacterial, antioxidant, anti-inflammatory, antiviral, anthelmintic, and anticancer activities. However, despite this pharmacological potential, research on the species remains limited, particularly regarding its phytochemistry in understudied plant parts, antivirulence properties, toxicity thresholds, pharmacokinetics, mechanisms of action, ecological distribution, and sustainable harvesting. Existing studies show that ethanol, methanol, and acetone extracts frequently demonstrate stronger bioactivities, likely due to their higher phenolic and flavonoid content. Anthelmintic investigations, though few, report moderate to high efficacy that supports traditional use, while available toxicity assessments indicate general safety at moderate concentrations. These biological effects appear to result from synergistic interactions among phytochemicals such as flavonoids, anthraquinones, chromones, and sterol glycosides. Compared with well-studied *Senna* species such as *S. alata, S. occidentalis*, and *S. italica*, the evidence base for *S. petersiana* remains comparatively sparse, highlighting clear knowledge gaps that need to be addressed to fully substantiate its therapeutic value.

## 7. Recommendation

Future research on *S. petersiana* should focus on conducting comprehensive in vivo studies to assess the pharmacological efficacy, pharmacokinetics, and safety profiles of its crude extracts and isolated compounds. Such studies are essential for translating in vitro bioactivities into clinically relevant outcomes. Additionally, bioassay-guided isolation of bioactive compounds must be prioritized to identify and characterize the specific compounds responsible for the observed pharmacological effects. Subsequent investigations should rigorously evaluate these isolated compounds for their bioactivities and toxicological properties to establish their therapeutic potential and safety margins. Furthermore, exploring interactions between isolated phytochemicals and conventional pharmaceutical agents is necessary to determine potential synergistic or antagonistic effects that may enhance efficacy or reduce toxicity. Studies should also investigate the phytochemistry of understudied plant parts and assess the species’ ecological distribution, cultivation potential for industrial applications, and sustainable harvesting practices to support responsible utilization. These approaches are critical to substantiating the medicinal value of S. petersiana and facilitating its integration into evidence-based therapeutics.

## Data Availability

All data are available on paper.
